# Dengue virus serotype 2 (DENV-2) outbreak, French Polynesia, 2019

**DOI:** 10.2807/1560-7917.ES.2019.24.29.1900407

**Published:** 2019-07-18

**Authors:** Maite Aubry, Mihiau Mapotoeke, Anita Teissier, Tuterarii Paoaafaite, Elsa Dumas-Chastang, Marine Giard, Van-Mai Cao-Lormeau

**Affiliations:** 1 Institut Louis Malardé, Papeete, Tahiti, French Polynesia; 2 Direction de la Santé de la Polynésie française, Papeete, Tahiti, French Polynesia

**Keywords:** Dengue virus, serotype 2, outbreak, French Polynesia, Pacific

## Abstract

In 1996–97, the last dengue virus serotype 2 (DENV-2) outbreak occurred in French Polynesia. In February 2019, DENV-2 infection was detected in a traveller from New Caledonia. In March, autochthonous DENV-2 infection was diagnosed in two residents. A DENV-2 outbreak was declared on 10 April with 106 cases as at 24 June. Most of the population is not immune to DENV-2; a large epidemic could occur with risk of imported cases in mainland France.

From March 2019 onwards, autochthonous dengue serotype 2 (DENV-2) infections have been detected in French Polynesia, an overseas collectivity of France in the South Pacific comprising ca 280,000 inhabitants and over 100 islands. On 10 April, French Polynesia public health authorities declared the beginning of the outbreak, 22 years after the last reported DENV-2 outbreak in 1996–97. Here, we describe the factors that could affect the magnitude of the outbreak and facilitate the spread of DENV-2 from French Polynesia to endemic and non-endemic areas, including European countries.

## Detection of imported and autochthonous cases of DENV-2 infections in French Polynesia in 2019

On 10 February 2019, a DENV-2 infection was diagnosed on Tahiti island (Society archipelago, French Polynesia) in a traveller from New Caledonia (French territory in the south-west Pacific), where a DENV-2 outbreak was ongoing [1]. The traveller arrived 1 day before symptom onset. Two days after the case was confirmed, vector control measures (insecticide spraying and destruction of breeding sites) were implemented at all locations that were visited by the case in Tahiti. In addition, the French Polynesia public health authorities enhanced surveillance to quickly detect any subsequent DENV-2 cases. No additional case was detected until two inhabitants of the same neighbourhood in Papeete (Tahiti) tested positive for DENV-2 on 18 and 29 March, respectively; neither case had travelled abroad within 2 weeks.

**Figure 1:**
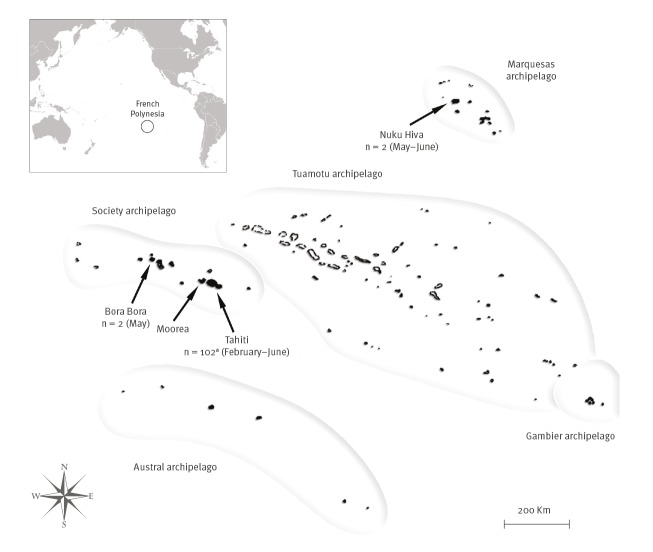
Map of cases of DENV-2 infection reported, French Polynesia, 10 February–24 June 2019 (n = 106)

On 10 April 2019, the public health authorities declared an outbreak as two separate clusters of DENV-2 cases, with no geographical or epidemiological link, had been confirmed on Tahiti island. As at 24 June, 102 DENV-2 cases were reported on Tahiti island; one case had just returned from Moorea island (Society archipelago) where the infection might have occurred; two other cases were imported from New Caledonia in February and April. DENV-2 infection was also detected in two residents from Nuku Hiva island (Marquesas archipelago) and two from Bora Bora island (Society archipelago) (Figure 1).

DENV-2: dengue virus serotype 2.

^a^ Among the 102 cases of DENV-2 infection reported on Tahiti, two were imported from New Caledonia and another had recently returned from Moorea where infection might have occurred.

Five archipelagos (Austral, Gambier, Marquesas, Society and Tuamotu) of French Polynesia are delineated by grey lines. Inset map at upper left shows location of French Polynesia in Pacific Ocean (black circle).

Between 10 February and 24 June 2019, fourplex real-time RT-PCR assay with serotype-specific primers and probes [2] was used to confirm DENV-2 infection in patients with symptoms suggestive of dengue (sudden high fever with headache, arthralgia and/or myalgia) in French Polynesia. Cases were defined as described in box.

Box **Case definition for dengue, French Polynesia, February–June 2019**

**Confirmed case:** Patients tested positive for dengue NS1 antigen (serotype unknown) and/or tested positive by real time RT-PCR (serotype known).

**Probable case:** Patients tested positive for anti-dengue IgM.

**Possible case:** Persons with dengue-like symptoms reported by a sentinel network of public and private practitioners distributed across French Polynesia.

In total, 248 dengue-like syndromes were reported; 63 patients tested positive for anti-dengue IgM; four patients tested positive for dengue NS1 antigen; 106 patients tested positive for DENV-2 and 225 patients tested positive for DENV-1. Among the patients diagnosed with DENV-2 or DENV-1 infection 69% and 42% were aged less than 20 years, 43% and 57% were males and 8% and 6% were hospitalised, respectively; no severe symptoms were reported in any case. Demographic and clinical characteristics of the DENV-2 and DENV-1 cases are shown in Table.

**Table:** Demographical and clinical characteristics of the DENV-2 (n=106) and DENV-1 (n=225) cases reported, French Polynesia, 10 February–24 June 2019

Characteristics	DENV-2		DENV-1	
	n = 106	%	n = 225	%
**Age group (years)**				
< 20	73	69	95	42
≥ 20	33	31	130	58
**Sex**				
Male	46	43	127	57
Female	60	57	98	43
**Outcome**				
Hospitalisation	8	8	13	6
Severe cases^a^	0	0	0	0

DENV-1: dengue virus serotype 1; DENV-2: dengue virus serotype 2.

^a^ Severe cases are defined as patients with at least one of the following symptoms: severe plasma leakage, severe haemorrhage and/or severe organ impairment.

## Phylogenetic analysis

The complete envelope gene of DENV-2 strains isolated in 2019 from five inhabitants of Tahiti, including three locally acquired infections (GenBank accession numbers: MK905539, MK905540 and MK905541) and two imported cases from New Caledonia (MK905538 and MK905542) were sequenced as previously described [3].

**Figure 2:**
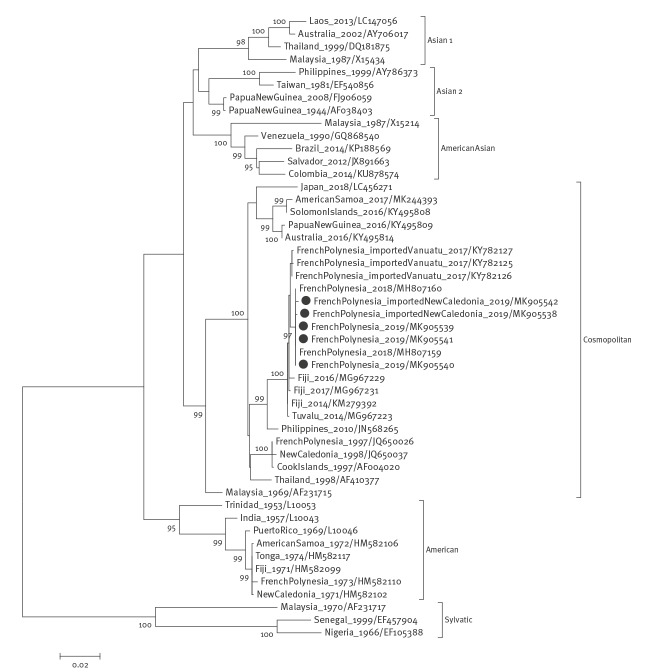
Phylogenetic analysis of DENV-2 strains isolated, French Polynesia, 2019 (n = 5)

Phylogenetic analysis showed that all DENV-2 strains collected in Tahiti belonged to the Cosmopolitan genotype and were more closely related to other strains isolated in French Polynesia in 2017 and 2018 (KY782125, KY782126, KY782127, MH807159 and MH807160), with percentages of homology ranging from 99.5% to 100% (Figure 2). The French Polynesia strains belonged to the same cluster as DENV-2 strains isolated in Tuvalu in 2014 (MG967223) and in Fiji during 2014–17 (KM279392, MG967229 and MG967231), with percentages of homology of more than 99.4%. Strains isolated in other Pacific islands such as the Solomon Islands in 2016 (KY495808) and the American Samoa in 2017 (MK244393) were more diverse with nt identity at 95.6% and 95.5%, respectively. The cluster including strains from French Polynesia, Tuvalu and Fiji and the cluster including strains from other Pacific islands were genetically closer to DENV-2 strains from Philippines (JN568265) and Australia (KY495814), respectively.

The phylogenetic tree was constructed using the MEGA7 software [17]. The evolutionary history was inferred by using the Maximum Likelihood method based on the Kimura 2-parameter model [18]. The percentage of trees in which the associated taxa clustered together is shown for values over 90 next to the branches (1,000 replicates). Each strain is labelled by country _ date of origin/GenBank accession number. The DENV-2 strains isolated in French Polynesia in 2019 are marked with a black circle.

## Discussion

In French Polynesia, the first outbreak of known DENV serotype occurred in 1944 [4]. Each of the four serotypes of DENV has caused several monotypic epidemics until 2013 [5] when an outbreak involving two different serotypes (DENV-1 and DENV-3) was reported [6], concomitantly to the transmission of Zika virus during 2013–14 [7] and then chikungunya virus during 2014–15 [8]. While DENV-3 stopped being detected in December 2014 [5], DENV-1 transmission was still reported as at 24 June 2019 [9]. During the period of monotypic endemic transmission of DENV-1, DENV-2 infection was detected in February 2017 in Tahiti from three travellers from Vanuatu participating in a soccer contest [3] and then in June 2018 in two residents of Raiatea island (Society archipelago) that had not recently travelled abroad [9]. Before the detection of those cases, the last DENV-2 epidemic had occurred in 1996–97 and autochthonous DENV-2 infection was reported for the last time in December 2000 [5].

The genetic similarity between DENV-2 strains isolated from patients infected in French Polynesia and in New Caledonia in 2019 suggests that DENV-2 was introduced into French Polynesia by travellers from New Caledonia. The first autochthonous DENV-2 infection reported in French Polynesia occurred 39 days after the detection of an imported case from New Caledonia, suggesting either DENV-2 was silently transmitted on Tahiti island during this period, or autochthonous transmission resulted from subsequent undetected viral introduction from New Caledonia. Tourist exchanges are frequent within the Pacific region and the introduction of DENV strains into French Polynesia from other Pacific islands, notably from New Caledonia, has been already reported [10,11]. In addition to New Caledonia, other Pacific islands (including Palau, Solomon Islands, Vanuatu, Fiji and American Samoa) have recently reported DENV-2 outbreaks [3]. Phylogenetic analysis of DENV-2 strains collected in French Polynesia, Tuvalu, Fiji, Solomon Islands and American Samoa revealed that two distinct lineages have circulated in the Pacific region since 2014, one likely originated from south-east Asia and the other from Australia. These findings could suggest that the Pacific islands are exposed to viral introductions from the rest of the world and there is the potential for onward spread.

The last DENV-2 outbreak in French Polynesia was reported more than 20 years ago. It has been shown that this time period was necessary for this serotype to re-emerge, as a sufficient proportion of the non-immune hosts has increased [3,6]. Serosurveys conducted in 2014 and 2018 in schoolchildren from Tahiti aged between 6 and 16 years found that none of them were immune against DENV-2, as they were all born after the last outbreak [12,13]. Serosurveys conducted in the general population from the five archipelagos in 2014 and from Tahiti and Moorea in 2015, showed that levels of population immunity against DENV-2 (51% and 18%, respectively) were lower than for the other serotypes (respectively 88% and 80% for DENV-1, 67% and 55% for DENV-3, and 61% and 42% for DENV-4) [12]. Altogether, those findings support the existence of a high risk for a large DENV-2 outbreak to occur in French Polynesia.

Despite multiple importations and autochthonous transmission of DENV-2 detected in French Polynesia during 2017–18, no infection was reported until 2019. Concomitant circulation of DENV-1 could have played a negative role in the transmission dynamics of DENV-2, as there may be competition between different serotypes for transmission by the mosquito host [14]. Another possible explanation for the absence of transmission of DENV-2 was a drier and colder season between April and October, which might have had a negative impact on mosquito density and vectorial capacity for transmission of the virus [15].

Despite the implementation of vector control, surveillance and prevention measures by the French Polynesia public health authorities, viral transmission could not be stopped and cases were reported on other islands shortly after the detection of the first imported and autochthonous DENV-2 infection in Tahiti. Given the frequency of air and sea links between Tahiti and the other islands, it is possible that DENV-2 could rapidly spread across French Polynesia. In addition, due to the frequent air travel exchanges between French Polynesia and non-endemic continental countries e.g. mainland France, DENV-2 could be introduced into these countries and cause outbreaks during the summer, which is the most favourable season for mosquito-borne transmission in temperate countries. For example, a DENV-1 outbreak following an imported case from French Polynesia was reported in South of France August–September 2015 [16]. Consequently, countries where competent vectors are prevalent need to be alerted to the risk of importation of DENV-2 from French Polynesia.
